# Supplementary material for: Discovery of a small-molecule inhibitor that traps Polθ on DNA and synergizes with PARP inhibitors
Source: Nat Commun. 2024 Apr 5;15:2862. doi: 10.1038/s41467-024-46593-1 (PMC10997755; doi:10.1038/s41467-024-46593-1)
Supplement: Supplementary file 1 — Supplementary Information [file 41467_2024_46593_MOESM1_ESM.pdf]

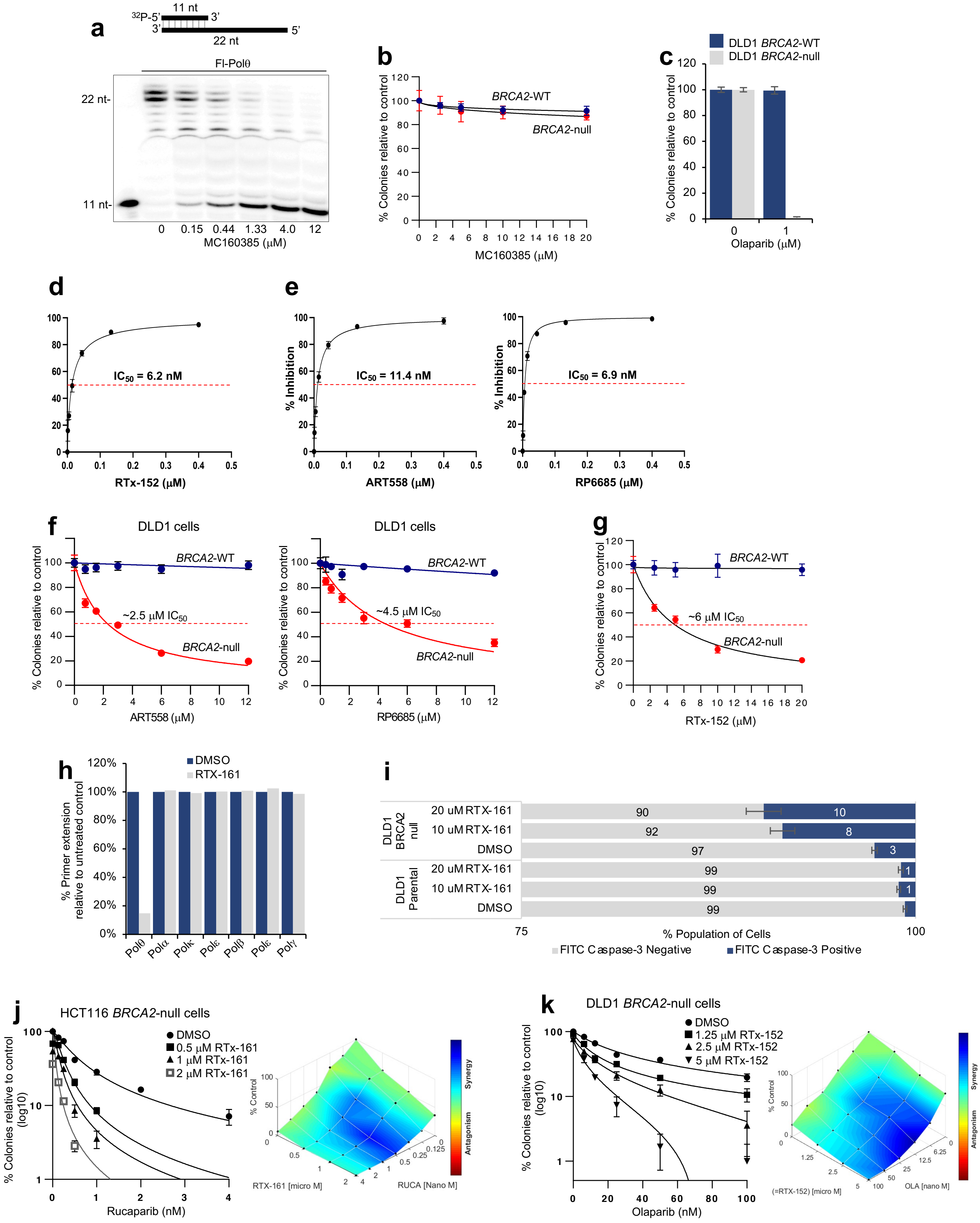

a
11 nt
32P-5'
3'
3'
5'
22 nt
Fl-Polθ
22 nt-
11 nt-
0 0.15 0.44 1.33 4.0 12
MC160385 (μM)
b
% Colonies relative to control
BRCA2-WT
BRCA2-null
MC160385 (μM)
c
DLD1 BRCA2-WT
DLD1 BRCA2-null
% Colonies relative to control
Olaparib (μM)
d
IC50 = 6.2 nM
RTx-152 (μM)
e
% Inhibition
IC50 = 11.4 nM
ART558 (μM)
IC50 = 6.9 nM
RP6685 (μM)
f
DLD1 cells
% Colonies relative to control
BRCA2-WT
~2.5 μM IC50
BRCA2-null
ART558 (μM)
DLD1 cells
BRCA2-WT
~4.5 μM IC50
BRCA2-null
RP6685 (μM)
g
BRCA2-WT
~6 μM IC50
BRCA2-null
RTx-152 (μM)
h
DMSO
RTX-161
% Primer extension relative to untreated control
Polθ Polα Polκ Polε Polβ Polε Polγ
i
DLD1 BRCA2 null
20 uM RTX-161 90 10
10 uM RTX-161 92 8
DMSO 97 3
DLD1 Parental
20 uM RTX-161 99 1
10 uM RTX-161 99 1
DMSO 99
75 % Population of Cells 100
FITC Caspase-3 Negative
FITC Caspase-3 Positive
j
HCT116 BRCA2-null cells
DMSO
0.5 μM RTx-161
1 μM RTx-161
2 μM RTx-161
% Colonies relative to control (log10)
Rucaparib (nM)
% Control
Synergy
Antagonism
RTX-161 [micro M]
RUCA [Nano M]
k
DLD1 BRCA2-null cells
DMSO
1.25 μM RTx-152
2.5 μM RTx-152
5 μM RTx-152
% Colonies relative to control (log10)
Olaparib (nM)
% Control
Synergy
Antagonism
(=RTX-152) [micro M]
OLA [nano M]

Supplementary Fig. 1 Supplemental assays evaluating the biochemical and cellular activities of Polθi.

a, Denaturing gel showing MC160385 inhibition of full-length Polq (Fl-Polq) DNA/DNA primer extension activity.

b, Scatter plot showing failure of MC160385 to induce synthetic lethality in BRCA2-null DLD1 cells. Data represent mean from 3 technical replicates. +/-s.d.

c, Bar plot showing induction of synthetic lethality by 1 μM Olaparib in BRCA2-null DLD1 cells. Data represent mean from 3 technical replicates. +/-s.d.

d, Scatter plot showing RTx-152 inhibition curve of Polθ-pol DNA synthesis activity. Data represent mean. n = 3 +/-s.d. IC_{50} = 6.2 nM.

e, Scatter plots showing ART558 (left) and RP6685 (right) inhibition curves of Polθ-pol DNA synthesis activity. Data represent mean. n = 3 +/-s.d. IC_{50} values are indicated.

f, Scatter plots showing clonogenic survival of the indicated *BRCA2*-WT and *BRCA2*-null DLD1 cells following treatment with the indicated concentrations of ART588 (left) and RP6685 (right). Data represent mean from n = 3 biological replicates. +/-s.d.

g, Scatter plot showing RTx-152 induction of synthetic lethality in *BRCA2*-null DLD1 cells. (n = 1) Data represent mean from 3 technical replicates. +/-s.d.

h, Bar plot showing % primer extension relative to untreated control by the indicated Pols in the presence of DMSO (blue) or RTx-161 (grey). Data represent mean from 2 technical replicates.

i, Bar plot showing % population of Caspase positive and negative BRCA2-null and BRCA2-WT DLD1 cells following treatment with DMSO or the indicated concentrations of RTx-161. Data represent mean from n = 3 biological replicates performed in triplicate. +/-s.d.

j, Scatter plots showing clonogenic survival of the indicated cell lines following treatment with the indicated concentrations Olaparib and RTx-161 (h) or RTx-152 (i) relative to DMSO controls (left). Percentage of colonies are normalized to DMSO treated cells (DMSO = 100%). Data represent mean from 3 technical replicates. +/-s.d. Synergy plots generated by Combenefit (right).

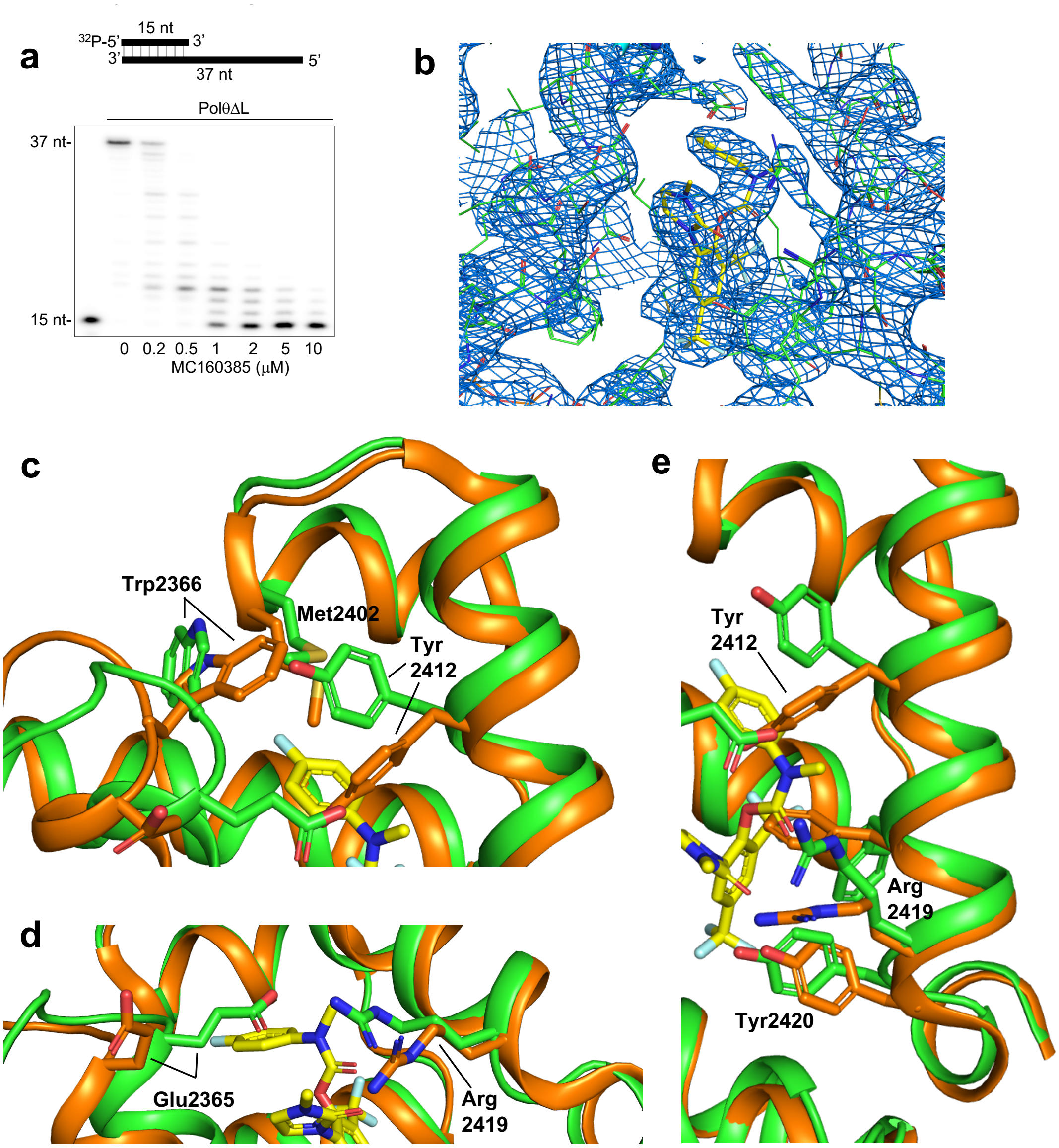

Supplementary Fig. 2 Supplemental figure for X-ray crystallography

a, Denaturing gel showing that Polθi MC160385 inhibits PolθΔL primer extension.

b, Electron density map of inhibitor binding site. Protein line model is in green, and inhibitor stick model is in yellow. 2mFo-DFc map visualized at contour level 1.5 sigma (blue).

c-e, Closeups of RTx-152 inhibitor binding pocket highlighting interacting residues and their movements upon RTx-152 binding. Green and orange represent RTx-152 bound and unbound (PDB 4x0q) structures, respectively.

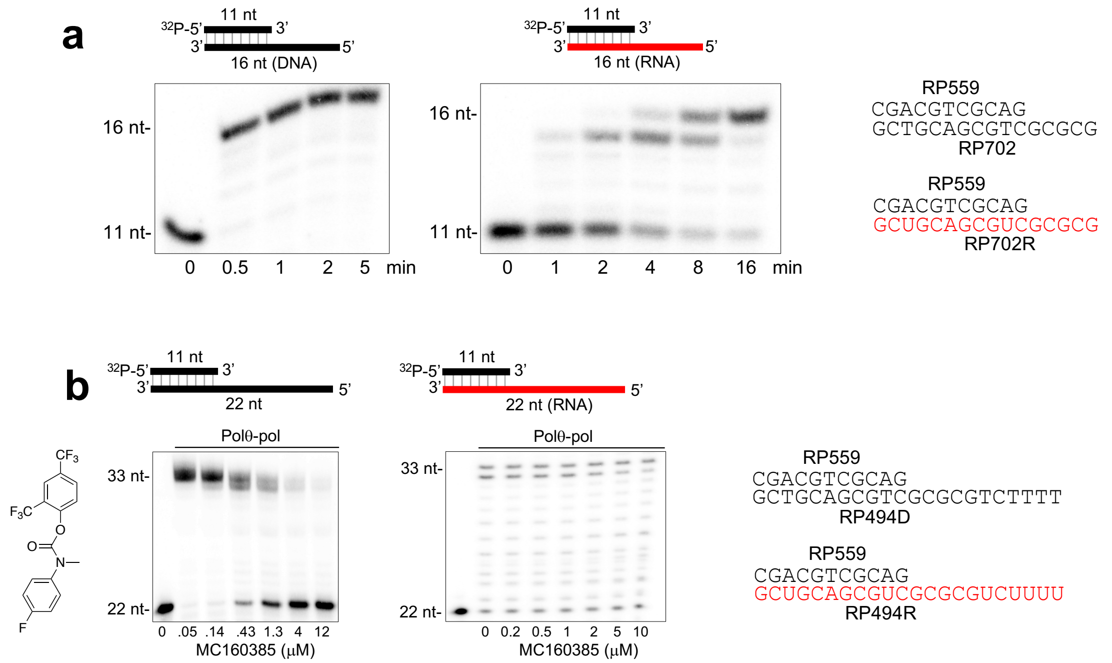

Supplementary Fig. 3. Control assays for Polθ-pol activity on DNA/DNA and DNA/RNA substrates.
a, Denaturing gels showing time courses of Polθ-pol primer extension on the indicated nucleic-acid primer templates (left, center). Schematic of DNA/DNA and DNA/RNA primer-template sequences (right). DNA, black type. RNA, red type.

b, Denaturing gels showing Polθ-pol primer extension on the indicated nucleic-acid primer templates in the presence of the indicated concentrations of Polθi MC160385 (left, center). Schematic of DNA/DNA and DNA/RNA primer-template sequences (right). DNA, black type. RNA, red type.

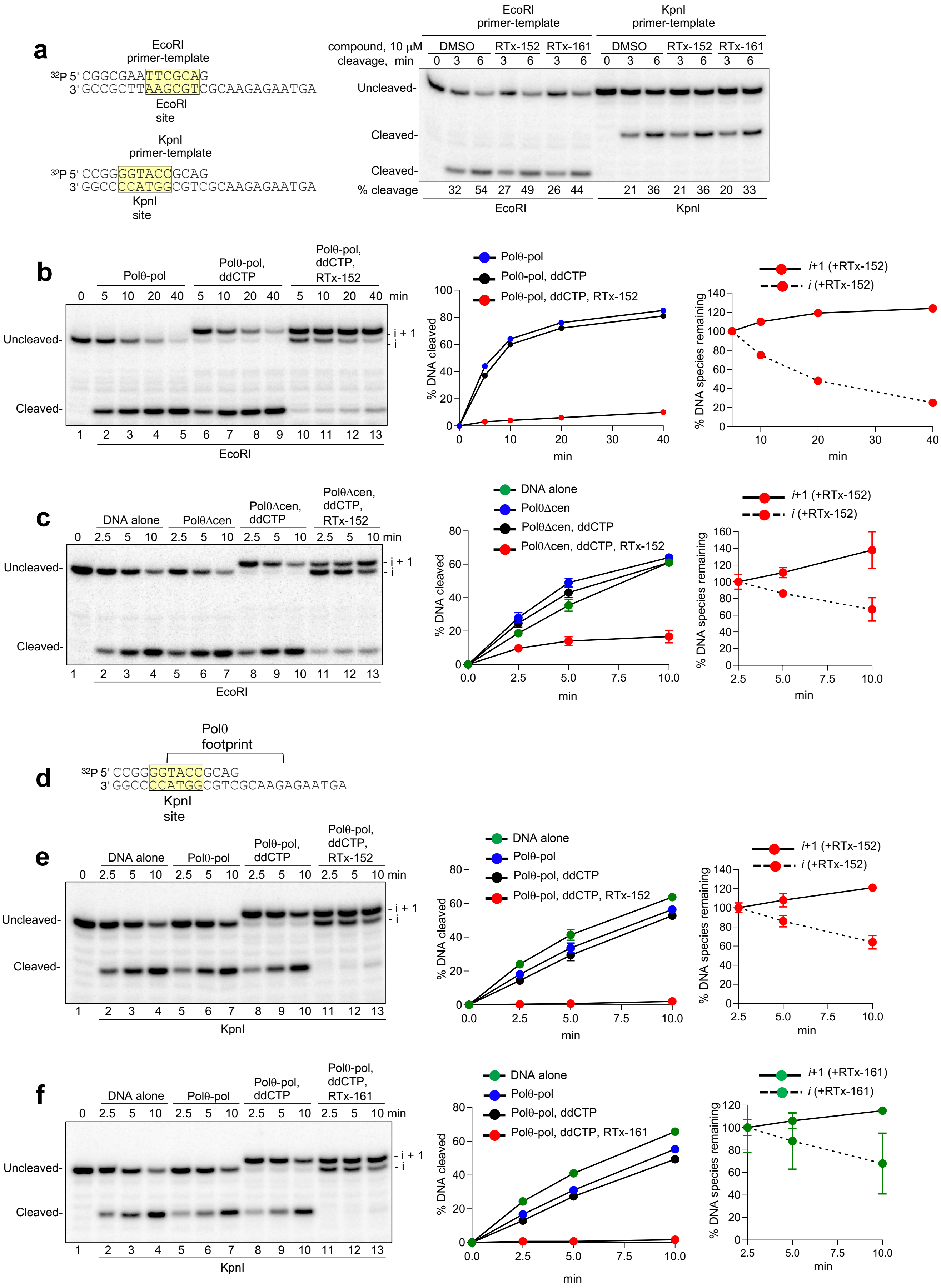
a
EcoRI
primer-template
32P 5' CGGCGAATTCGCAG
3' GCCGCTTAAGCGTCGCAAGAGAATGA
EcoRI
site
KpnI
primer-template
32P 5' CCGGGGTACCGCAG
3' GGCCCCATGGCGTCGCAAGAGAATGA
KpnI
site
EcoRI
primer-template
KpnI
primer-template
compound, 10 µM
DMSO
RTx-152
RTx-161
cleavage, min
0 3 6 3 6 3 6 0 3 6 3 6 3 6
Uncleaved-
Cleaved-
Cleaved-
% cleavage
32 54 27 49 26 44
21 36 21 36 20 33
EcoRI
KpnI
b
Polθ-pol
Polθ-pol, ddCTP
Polθ-pol, ddCTP, RTx-152
0 5 10 20 40 5 10 20 40 5 10 20 40 min
Uncleaved-
Cleaved-
- i + 1
- i
1 2 3 4 5 6 7 8 9 10 11 12 13
EcoRI
Polθ-pol
Polθ-pol, ddCTP
Polθ-pol, ddCTP, RTx-152
% DNA cleaved
min
i+1 (+RTx-152)
i (+RTx-152)
% DNA species remaining
min
c
DNA alone
PolθΔcen
PolθΔcen, ddCTP
PolθΔcen, ddCTP, RTx-152
0 2.5 5 10 2.5 5 10 2.5 5 10 2.5 5 10 min
Uncleaved-
Cleaved-
- i + 1
- i
1 2 3 4 5 6 7 8 9 10 11 12 13
EcoRI
DNA alone
PolθΔcen
PolθΔcen, ddCTP
PolθΔcen, ddCTP, RTx-152
% DNA cleaved
min
i+1 (+RTx-152)
i (+RTx-152)
% DNA species remaining
min
d
Polθ
footprint
32P 5' CCGGGGTACCGCAG
3' GGCCCCATGGCGTCGCAAGAGAATGA
KpnI
site
e
DNA alone
Polθ-pol
Polθ-pol, ddCTP
Polθ-pol, ddCTP, RTx-152
0 2.5 5 10 2.5 5 10 2.5 5 10 2.5 5 10 min
Uncleaved-
Cleaved-
- i + 1
- i
1 2 3 4 5 6 7 8 9 10 11 12 13
KpnI
DNA alone
Polθ-pol
Polθ-pol, ddCTP
Polθ-pol, ddCTP, RTx-152
% DNA cleaved
min
i+1 (+RTx-152)
i (+RTx-152)
% DNA species remaining
min
f
DNA alone
Polθ-pol
Polθ-pol, ddCTP
Polθ-pol, ddCTP, RTx-161
0 2.5 5 10 2.5 5 10 2.5 5 10 2.5 5 10 min
Uncleaved-
Cleaved-
- i + 1
- i
1 2 3 4 5 6 7 8 9 10 11 12 13
KpnI
DNA alone
Polθ-pol
Polθ-pol, ddCTP
Polθ-pol, ddCTP, RTx-161
% DNA cleaved
min
i+1 (+RTx-161)
i (+RTx-161)
% DNA species remaining
min

Supplementary Fig. 4 Control assays for Polθi DNA trapping.

a, Schematic of the DNA/DNA primer-template used for the endonuclease protection assays (left). Denaturing gel showing that EcoRI and KpnI are resistant to inhibition by RTx-161 and RTx-152 (right).

b,c, Denaturing gel showing EcoRI cleavage of the DNA/DNA primer-template at the indicated times following pre-incubation with or without Polθ-pol (b) or PolθΔcen (c), and pre-incubation with ddCTP and RTx-152 as indicated (left). Scatter plot showing the relative rates of EcoRI cleavage following pre-incubation with or without Polθ-pol (b) or PolθΔcen (c), ddCTP and Polθi as indicated (middle). Data represent mean. n = 3 +/-s.d. Scatter plot showing the relative rates of disappearance of the DNA/DNA species within the i and i + 1 complexes (right). Data represent mean. n = 3 +/-s.d. i, DNA within pre-catalytic complexes. i + 1, DNA within post-catalytic complexes.

d, Schematic of DNA/DNA with KpnI recognition site.

e,f, Denaturing gel showing KpnI cleavage of the DNA/DNA primer-template at the indicated times following pre-incubation with or without Polθ-pol, and pre-incubation with ddCTP and RTx-152 or RTx-161 as indicated (left). Scatter plot showing the relative rates of KpnI cleavage following pre-incubation with or without Polθ-pol, ddCTP and RTx-152 or RTx-161 as indicated (middle). Data represent mean. n = 3 +/-s.d. Scatter plot showing the relative rates of disappearance of the DNA/DNA species within the i and i + 1 complexes (right). Data represent mean. n = 3 +/-s.d. i, DNA within pre-catalytic complexes. i + 1, DNA within post-catalytic complexes.

Synthesis of Polθi.

RTx-152

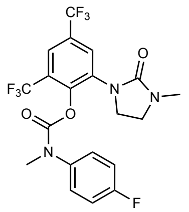

Step 1: (4-Fluorophenyl)(methyl)carbamic chloride

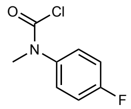

To a solution of 4-fluoro-N-methylaniline (0.58 g, 4.66 mmol) and pyridine (0.73 g, 9.34 mmol) in 12 mL of dichloromethane at 0 °C, triphosgene (0.69 g, 2.33 mmol) dissolved in 6 mL dichloromethane was added dropwise under inert atmosphere. The reaction was stirred at ambient temperature (room temperature) for 2 h. The reaction mixture was diluted with 20 mL dichloromethane and extracted with 20 mL 1N HCl. The organic layer was separated, dried over with anhydrous sodium sulfate, filtered and concentrated to a solid under reduced pressure. ^{1}H NMR (400 MHz, $CDCl_3$) δ 7.15 (m, 2H), 7.04 (m, 2H), 3.3 (s, 3H).; MS(ESI): m/z 188.0 [$(M+H)^+$].

Step 2: 2-iodo-4,6-bis(trifluoromethyl)phenol

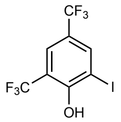

A solution of 2,4-bis(trifluoromethyl)phenol (1.00 g, 4.35 mmol) in THF:H_2O (3:1, 24 mL) was cooled to 0°C in an ice bath under nitrogen. Iodine (1.18 g, 4.64 mmol) and Na_2CO_3 (491 mg, 4.64 mmol) were added sequentially. The ice bath was removed and the reaction was allowed to warm to RT with stirring overnight. The reaction solution was cooled to 0°C in an ice bath, quenched with saturated aqueous sodium metabisufite and stirred at 0°C until all of the solution turned yellow in color. This mixture was extracted with EtOAc (3X). The combined organic extracts were washed with water and brine, dried over anhydrous sodium sulfate and concentrated. The crude product was purified by column chromatography on silica gel using a gradient solvent system of 0 to 10% of ethyl acetate in hexanes to afford the titled compound as colorless crystalline solid (711 mg, 46%). ^{1}H NMR (400 MHz, $CDCl_3$) δ 8.10 (s, 1H), 7.82 (s, 1H), 6.15 (s, 1H).

Step 3: 2-iodo-4,6-bis(trifluoromethyl)phenyl (4-fluorophenyl)(methyl)carbamate

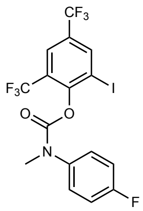

2-iodo-4,6-bis(trifluoromethyl)phenol (570 mg, 1.60 mmol) and (4-fluorophenyl)(methyl)carbamic chloride (751 mg, 4.00 mmol) were dissolved into anhydrous pyridine (10 mL).This solution was stirred at 90°C for 4 hours. The reaction was cooled to RT and concentrated down. The residual solid was partitioned between EtOAc and 1N aqueous HCl. The aqueous phase was separated and extracted with EtOAc twice. The combined organic extracts were washed with brine, dried over anhydrous sodium sulfate and concentrated. The crude product was purified by column chromatography on silica gel using a gradient solvent system of 0 to 20% of ethyl acetate in hexanes to afford the titled compound as a yellow oil (683 mg, 84%). ^{1}H NMR (400 MHz, $CDCl_3$) δ 8.21-8.27 (1H), 7.84-7.92 (1H), 7.39 (m, 2H), 7.12 (m, 2H), 3.38-3.56 (3H); ESIMS: *m/z* 508.0 [$(M+H)^+$]

Step 4:

2-iodo-4,6-bis(trifluoromethyl)phenyl (4-fluorophenyl)(methyl)carbamate (0.1972 mmol, 100 mg*), 1-* Methy*l*-2-imidazolidinone (0.39 mmol, 39 mg), copper (I) iodide (0.0961 mmol, 18.3 mg), cesium fluoride (0.3995 mmol, 60.7 mg), N,N'-dimethylethylenediamine (0.1972 mmol, 21.2 uL) and anhydrous powered potassium carbonate (0.3641 mmol, 50.3 mg) were added to degassed anhydrous 1,4-dioxane (5.0 mL) under nitrogen. The resulting suspension was stirred at 90°C overnight. The reaction was cooled to room temperature, filtered and the filtrate was concentrated down to yield a semi-solid. The crude product was purified by column chromatography on silica gel using a gradient solvent system of 0 to 100% of ethyl acetate in hexanes to afford the titled compound as a yellow oil (2 mg, 2%). ^{1}H NMR (400 MHz, $CDCl_3$) δ 7.86-7.7 (m,2H), 7.32 (m, 2H), 7.09 (m, 2H), 3.70(m, 2H), 3.51 (m, 2H), 3.45 (d, *J*= 44Hz, 3H), 2.92(s, 3H); ESIMS: *m/z* 480.14 [$(M+H)^+$] .

RTx-161

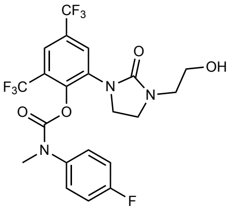

Step 1: (4-Fluorophenyl)(methyl)carbamic chloride

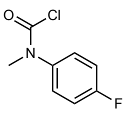

To a solution of 4-fluoro-N-methylaniline (0.58 g, 4.66 mmol) and pyridine (0.73 g, 9.34 mmol) in 12 mL

of dichloromethane at 0 °C, triphosgene (0.69 g, 2.33 mmol) dissolved in 6 mL dichloromethane was added dropwise under inert atmosphere. The reaction was stirred at ambient temperature (room temperature) for 2 h. The reaction mixture was diluted with 20 mL dichloromethane and extracted with 20 mL 1N HCl. The organic layer was separated, dried over with anhydrous sodium sulfate, filtered and concentrated to a solid under reduced pressure. ^{1}H NMR (400 MHz, $CDCl_3$) δ 7.15 (m, 2H), 7.04 (m, 2H), 3.3 (s, 3H).; MS(ESI): m/z 188.0 [$(M+H)^+$].

Step 2: 2-iodo-4,6-bis(trifluoromethyl)phenol

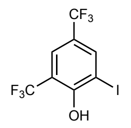

A solution of 2,4-bis(trifluoromethyl)phenol (1.00 g, 4.35 mmol) in THF:H_2O (3:1, 24 mL) was cooled to 0°C in an ice bath under nitrogen. Iodine (1.18 g, 4.64 mmol) and Na_2CO_3 (491 mg, 4.64 mmol) were added sequentially. The ice bath was removed and the reaction was allowed to warm to RT with stirring overnight. The reaction solution was cooled to 0°C in an ice bath, quenched with saturated aqueous sodium metabisufite and stirred at 0°C until all of the solution turned yellow in color. This mixture was extracted with EtOAc (3X). The combined organic extracts were washed with water and brine, dried over anhydrous sodium sulfate and concentrated. The crude product was purified by column chromatography on silica gel using a gradient solvent system of 0 to 10% of ethyl acetate in hexanes to afford the titled compound as colorless crystalline solid (711 mg, 46%). ^{1}H NMR (400 MHz, $CDCl_3$) δ 8.10 (s, 1H), 7.82 (s, 1H), 6.15 (s, 1H).

Step 3: 2-iodo-4,6-bis(trifluoromethyl)phenyl (4-fluorophenyl)(methyl)carbamate

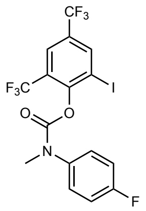

2-iodo-4,6-bis(trifluoromethyl)phenol (570 mg, 1.60 mmol) and (4-fluorophenyl)(methyl)carbamic chloride (751 mg, 4.00 mmol) were dissolved into anhydrous pyridine (10 mL).This solution was stirred at 90°C for 4 hours. The reaction was cooled to RT and concentrated down. The residual solid was partitioned between EtOAc and 1N aqueous HCl. The aqueous phase was separated and extracted with EtOAc twice. The combined organic extracts were washed with brine, dried over anhydrous sodium sulfate and concentrated. The crude product was purified by column chromatography on silica gel using a gradient solvent system of 0 to 20% of ethyl acetate in hexanes to afford the titled compound as a yellow oil (683 mg, 84%). ^{1}H NMR (400 MHz, $CDCl_3$) δ 8.21-8.27 (1H), 7.84-7.92 (1H), 7.39 (m, 2H), 7.12 (m, 2H), 3.38-3.56 (3H); ESIMS: *m/z* 508.0 [$(M+H)^+$]

Step 4

2-iodo-4,6-bis(trifluoromethyl)phenyl (4-fluorophenyl)(methyl)carbamate (0.1972 mmol, 100 mg*),* 1-(2-Hydroxyethyl-2-imidazolidinone (0.39 mmol, 51 mg), copper (I) iodide (0.0961 mmol, 18.3 mg), cesium fluoride (0.3995 mmol, 60.7 mg), N,N'-dimethylethylenediamine (0.1972 mmol, 21.2 uL) and anhydrous powered

potassium carbonate (0.3641 mmol, 50.3 mg) were added to degassed anhydrous 1,4-dioxane (5.0 mL) under nitrogen. The resulting suspension was stirred at 90°C overnight. The reaction was cooled to room temperature, filtered and the filtrate was concentrated down to yield a semi-solid. The crude product was purified by column chromatography on silica gel using a gradient solvent system of 0 to 100% of ethyl acetate in hexanes to afford the titled compound as a yellow oil (11 mg, 11%). ^{1}H NMR (400 MHz, $CDCl_3$) δ 7.81 (m,2H), 7.32 (m, 2H), 7.09 (m, 2H), 3.85(s, 3H), 3.70 (s, 1H), 3.61 (m, 2H), 3,47(m, 4H), 3.36(m,2H); ESIMS: *m/z* 510.48 [$(M+H)^+$]
