# Supplementary material for: Discovery of a small-molecule inhibitor that traps Polθ on DNA and synergizes with PARP inhibitors
Source: Nat Commun. 2024 Apr 5;15:2862. doi: 10.1038/s41467-024-46593-1 (PMC10997755; doi:10.1038/s41467-024-46593-1)
Supplement: Supplementary file 3 — Description of Additional Supplementary Files [file 41467_2024_46593_MOESM3_ESM.pdf]

Description of Additional Supplementary Files

File Name: Supplementary Movie 1
Description: Induced fit binding mechanism of RTx-152. The open to closed conformational change of the fingers subdomain is rendered followed by RTx-152 binding and subsequent residue movement around RTx-152 within the hydrophobic binding pocket formed in the closed conformation.

File Name: Supplementary Movie 2
Description: An additional view of the Induced fit binding mechanism of RTx-152. The open to closed conformational change of the fingers subdomain is rendered followed by RTx-152 binding and subsequent residue movement around RTx-152 within the hydrophobic binding pocket formed in the closed conformation.
