# Supplementary material for: Discovery of a small-molecule inhibitor that traps Polθ on DNA and synergizes with PARP inhibitors
Source: Nat Commun. 2024 Apr 5;15:2862. doi: 10.1038/s41467-024-46593-1 (PMC10997755; doi:10.1038/s41467-024-46593-1)
Supplement: Supplementary file 6 — Reporting Summary [file 41467_2024_46593_MOESM6_ESM.pdf]

nature portfolio

Corresponding author(s): Richard Pomerantz

Last updated by author(s): Feb 26, 2024

Reporting Summary

Nature Portfolio wishes to improve the reproducibility of the work that we publish. This form provides structure for consistency and transparency in reporting. For further information on Nature Portfolio policies, see our Editorial Policies and the Editorial Policy Checklist.

Statistics

For all statistical analyses, confirm that the following items are present in the figure legend, table legend, main text, or Methods section.

n/a	Confirmed	
☐	☒	The exact sample size (*n*) for each experimental group/condition, given as a discrete number and unit of measurement
☐	☒	A statement on whether measurements were taken from distinct samples or whether the same sample was measured repeatedly
☐	☒	The statistical test(s) used AND whether they are one- or two-sided<br>*Only common tests should be described solely by name; describe more complex techniques in the Methods section.*
☒	☐	A description of all covariates tested
☐	☒	A description of any assumptions or corrections, such as tests of normality and adjustment for multiple comparisons
☐	☒	A full description of the statistical parameters including central tendency (e.g. means) or other basic estimates (e.g. regression coefficient) AND variation (e.g. standard deviation) or associated estimates of uncertainty (e.g. confidence intervals)
☐	☒	For null hypothesis testing, the test statistic (e.g. *F*, *t*, *r*) with confidence intervals, effect sizes, degrees of freedom and *P* value noted<br>*Give P values as exact values whenever suitable.*
☒	☐	For Bayesian analysis, information on the choice of priors and Markov chain Monte Carlo settings
☒	☐	For hierarchical and complex designs, identification of the appropriate level for tests and full reporting of outcomes
☒	☐	Estimates of effect sizes (e.g. Cohen's *d*, Pearson's *r*), indicating how they were calculated

Our web collection on statistics for biologists contains articles on many of the points above.

Software and code

Policy information about availability of computer code

Data collection	JBluelce-Epics is the graphical user interface of the GM/CA beamline control system at the Advanced Photon Source. JBluelce-Epics was used for data-collection of the protein structure highlighted in this manuscript. This software was accessed on 11/22/2022 while operating beamline 23-ID-D.<br>Facscanto (Becton Dickinson, Chemidoc imaging system (Bio-rad)
Data analysis	HKL-2000 was used for the reduction and scaling of the data collected at the APS. The version number of HKL-2000 used was v722.<br><br>PHENIX was used for the phasing and refinement of the protein structure listed in this manuscript. The version of PHENIX used was 1.20.1-4487. Phenix's eLBOW and REEL programs were used to generate PDB and restrain files of the RTX-152 ligand.<br><br>winCoot was used for model building of the protein structure in between refinement rounds. The version of this software used is 0.9.8.7.<br><br>Combenefit" software version 2.021 was used to perform synergy analyses for drug combinations. GraphPad Prism 9 software was used to calculate ICS0. Microsoft Excel was used for analysis. Quantification was performed using ImageQuant TL software for invitro MMEJ assays.

For manuscripts utilizing custom algorithms or software that are central to the research but not yet described in published literature, software must be made available to editors and reviewers. We strongly encourage code deposition in a community repository (e.g. GitHub). See the Nature Portfolio guidelines for submitting code & software for further information.

Data

Policy information about availability of data

All manuscripts must include a data availability statement. This statement should provide the following information, where applicable:

- Accession codes, unique identifiers, or web links for publicly available datasets
- A description of any restrictions on data availability
- For clinical datasets or third party data, please ensure that the statement adheres to our policy

The X-ray crystallography structure of PolqDL-pol bound to DNA/DNA, RTx-152 and ddGMP was deposited in the Protein Data Bank under accession code PDB 8GD7 . Source data are provided with this paper.
Any other protocol and materials are available from the corresponding author on reasonable request.

Research involving human participants, their data, or biological material

Policy information about studies with human participants or human data. See also policy information about sex, gender (identity/presentation), and sexual orientation and race, ethnicity and racism.

Reporting on sex and gender	N/A
Reporting on race, ethnicity, or other socially relevant groupings	N/A
Population characteristics	N/A
Recruitment	N/A
Ethics oversight	N/A

Note that full information on the approval of the study protocol must also be provided in the manuscript.

Field-specific reporting

Please select the one below that is the best fit for your research. If you are not sure, read the appropriate sections before making your selection.

☒ Life sciences ☐ Behavioural & social sciences ☐ Ecological, evolutionary & environmental sciences

For a reference copy of the document with all sections, see nature.com/documents/nr-reporting-summary-flat.pdf

Life sciences study design

All studies must disclose on these points even when the disclosure is negative.

Sample size	Sample size were chosen according to the standards of the field for both cell based and invitro biochemical assay (at least three independent biological replicates for each condition per experiment unless otherwise stated). The sample size was determined based on our experimental observations and experiences in which such numbers could give reliable and reproducible results.
Data exclusions	We did not exclude any data from consideration.
Replication	Reported results were consistently replicated across multiple experiments with all replicates generating similar results. At least three replicates were used for all experiments unless otherwise stated. Representative graphs, gels and micrographs from these experiments are presented. Description of the number of replicates and error bars are included in the corresponding figure legends.
Randomization	No randomization was necessary for this study because all assays were performed in well controlled conditions. All samples in various assays were treated uniformly and same data analysis procedure was applied to all samples of the same experiment.
Blinding	Blinding is also not necessary because the results presented are based on quantitative analysis and did not require subjective judgment or interpretation or involved human biases.

Reporting for specific materials, systems and methods

We require information from authors about some types of materials, experimental systems and methods used in many studies. Here, indicate whether each material, system or method listed is relevant to your study. If you are not sure if a list item applies to your research, read the appropriate section before selecting a response.

Materials & experimental systems

n/a	Involved in the study
☐	☒ Antibodies
☐	☒ Eukaryotic cell lines
☒	☐ Palaeontology and archaeology
☒	☐ Animals and other organisms
☒	☐ Clinical data
☒	☐ Dual use research of concern
☒	☐ Plants

Methods

n/a	Involved in the study
☒	☐ ChIP-seq
☒	☐ Flow cytometry
☒	☐ MRI-based neuroimaging

Antibodies

Antibodies used

H2AX (p Ser 139) was detected using antibody (NOVUS #NB100-384) diluted 1:2000. Cleaved PARP was detected using antibody (Cell Signaling #9546) diluted 1:2,000. Gapdh was detected using Gapdh (14C10) rabbit monoclonal antibody (Cell Signaling Technology #2118) diluted 1:4000. Secondary antibodies -Goat anti-Rabbit IgG (H+L) HRP (Invitrogen #31466) or Goat anti-Mouse IgG (H+L) HRP (Invitrogen #31430)–tagged Secondary Antibody diluted 1:5,000. The primary antibody used for IF was rabbit anti-gamma H2AX [p Ser139] antibody (Bethyl Lab #A700-053) 1:500 dilution. The secondary antibody was Goat anti-Rabbit IgG (H+L) Secondary Antibody, DyLight 488 (Thermo #35552) 1:2000.

Validation

All antibodies have been validated by the respective manufacturer by demonstrating immunoblotting on cell extracts and /or inhibition of this signal with an immunizing peptide. In addition, antibodies have been validated in multiple publications.

Eukaryotic cell lines

Policy information about cell lines and Sex and Gender in Research

Cell line source(s)

U2OS cells with MMEJ reporter (EJ2-GFP) was a kind gift from Dr. Jeremy Stark (City of Hope) . DLD1 BRCA2 -/- and DLD1 Parental were obtained from Horizon discovery, Waterbeach, UK. HCT 116 BRCA2 -/- and HCT 116 Parental were obtained from Cancertools, London, UK. MEF BRCA1 -/- and MEF Parental was a kind gift from Dr. Neil Johnson (Fox chase Cancer Center). EUFA1341 PALB2 mut, PALB2 wildtype was a kind gift from Dr. Bing Xia (Rutgers University). MDA 436 BRCA1 mut and MDA 231 (used as wildtype control for MDA 436) cells were obtained from ATCC, Manassas, VA.

Authentication

None of the cell lines have been authenticated.

Mycoplasma contamination

Cell lines were routinely tested for mycoplasma contamination and no indication of contamination was observed.

Commonly misidentified lines
(See ICLAC register)

No commonly misidentified cell lines were used.

Plants

Seed stocks

Report on the source of all seed stocks or other plant material used. If applicable, state the seed stock centre and catalogue number. If plant specimens were collected from the field, describe the collection location, date and sampling procedures.

Novel plant genotypes

Describe the methods by which all novel plant genotypes were produced. This includes those generated by transgenic approaches, gene editing, chemical/radiation-based mutagenesis and hybridization. For transgenic lines, describe the transformation method, the number of independent lines analyzed and the generation upon which experiments were performed. For gene-edited lines, describe the editor used, the endogenous sequence targeted for editing, the targeting guide RNA sequence (if applicable) and how the editor was applied.

Authentication

Describe any authentication procedures for each seed stock used or novel genotype generated. Describe any experiments used to assess the effect of a mutation and, where applicable, how potential secondary effects (e.g. second site T-DNA insertions, mosiacism, off-target gene editing) were examined.
